# Aggregated occurrence records of invasive European frog-bit (*Hydrocharismorsus-ranae* L.) across North America

**DOI:** 10.3897/BDJ.10.e77492

**Published:** 2022-02-09

**Authors:** Sara E. Hansen, Blake C. Cahill, Rachel A. Hackett, Michael J. Monfils, Ryan T. Goebel, Shannon Asencio, Anna Monfils

**Affiliations:** 1 Central Michigan University Earth and Ecosystem Science Program, Mount Pleasant, Michigan, United States of America Central Michigan University Earth and Ecosystem Science Program Mount Pleasant, Michigan United States of America; 2 Michigan State University Extension Michigan Natural Features Inventory, Lansing, Michigan, United States of America Michigan State University Extension Michigan Natural Features Inventory Lansing, Michigan United States of America; 3 Central Michigan University Department of Biology, Mount Pleasant, Michigan, United States of America Central Michigan University Department of Biology Mount Pleasant, Michigan United States of America; 4 Agriculture and Agri-Food Canada National Collection of Vascular Plants, Ottawa, Ontario, Canada Agriculture and Agri-Food Canada National Collection of Vascular Plants Ottawa, Ontario Canada; 5 Central Michigan University Herbarium and Department of Biology, Mount Pleasant, Michigan, United States of America Central Michigan University Herbarium and Department of Biology Mount Pleasant, Michigan United States of America

**Keywords:** European frog-bit, Laurentian Great Lakes, invasive plants, aquatic ecosystems, wetlands, occurrence records, specimen data

## Abstract

**Background:**

European frog-bit (*Hydrocharismorsus-ranae* L.; EFB) is a free-floating aquatic plant invasive in Canada, the United States and India. It is native to Europe and northern and western Asia and is believed to have first been introduced to North America in Ottawa, Ontario in 1932. It has since spread by way of the St. Lawrence River and connected waterways to southern Ontario and Quebec and parts of the northern United States. Invasive European frog-bit occurs in freshwater coastal wetlands and inland waters, where it can form dense mats that have the potential to limit recreational and commercial use of waterways, alter water chemistry and impact native species and ecosystems. Data on the past and present distribution of this invasive species provide geospatial information that can be used to infer the pattern of invasion and inform management and monitoring targeted at preventing secondary spread. Our EFB dataset contains 12,037 preserved specimen and observation-based occurrence records, including 9,994 presence records spanning two Canadian provinces and ten U.S. states and 2,043 absence records spanning five U.S. states. The aggregated EFB dataset provides a curated resource that has been used to guide a Michigan management strategy and provide information for ongoing efforts to develop invasion risk assessments, species distribution models and decision-support tools for conservation and management.

**New information:**

Specimen-based and observation-based occurrence data were accessed through nine digital data repositories or aggregators and three primary sources. Twenty-six percent of the data are new records not previously published to a data repository or aggregator prior to this study. We removed duplicate data and excluded records with incorrect species identifications. Occurrence records without coordinates were georeferenced from recorded locality descriptions. Data were standardised according to Darwin Core. This aggregated dataset is the most complete account of EFB occurrence records in its North American invasive range.

## Introduction

European frog-bit (*Hydrocharismorsus-ranae* L.; EFB) is a free-floating aquatic plant, native to Europe and northern and western Asia and invasive to North America and India (Cook and Lüönd 1982, Catling et al. 2003, Ganie et al. 2016). In 1932, EFB was introduced as an ornamental at the Central Experimental Farm in Ottawa, Ontario, Canada (Minshall 1940). In 1939, it was first observed outside of cultivation in the nearby Rideau Canal (Minshall 1940). As of July 2021, EFB populations in Canada occur north of Ottawa into Quebec and north and south of Ottawa into other parts of Ontario. In 1974, EFB was first observed in the United States. A specimen was collected near the St. Lawrence River in St.Lawrence County, New York (Uhler 1974, Roberts et al. 1981). As of July 2021, EFB occurs in the U.S. states of Florida, Maine, Michigan, New Jersey, New York, Ohio, Pennsylvania, Vermont, Washington State and Wisconsin. A single occurrence of EFB was recorded in an unknown waterbody of Madison County, Florida via iNaturalist in February of 2021. As of July 2021, no new occurrences have been recorded in Florida. A voucher was made from an established population in Morris County, New Jersey in October 2013. The specimen is not available, but one record was created for the aggregated dataset from literature reporting this occurrence (Lamont et al. 2014). A small population of EFB was found near Meadow Lake in Snohomish County, Washington State in 1997 (Environmental Assessment Program 2018). The aggregated dataset contains eight specimen-based and two observation-based records in the same location of Washington as recently as 2016, which suggests this population is persisting, but not spreading. A population of EFB was recorded in a stream and adjacent drainage ditches in Oconto County, Wisconsin via iNaturalist in July 2021. In 2013, EFB was first observed in India in two wetlands of the Kashmir Himalaya (Ganie et al. 2016). Although the current distribution of EFB in this region is not yet known, there is concern that it may spread and dominate waterbodies and negatively impact ecosystem health and subsistence aquaculture (Masoodi 2021). As invasive EFB continues to spread and threaten waterways of North America and India, continued aggregation of occurrence records is crucial for monitoring and managing EFB and its impacts.

European frog-bit grows in rosettes and reproduces both sexually and asexually and can be found in Laurentian Great Lakes coastal wetlands, inland lakes and slow-moving streams and man-made waterbodies, such as ponds and roadside ditches (Catling and Dore 1982, Monfils et al. 2021). Fruit and viable seed production have been observed in both the native and invasive range (Arber 1920, Burnham 1998). Flowers are insect-pollinated; after fertilisation, female flowers recurve into the water where many-seeded berries ripen and dehisce (Cook and Lüönd 1982, Scribailo and Posluszny 1984). Greater than 90% of seeds collected from an invasive population in North America germinated in ex-situ trials (Cahill et al. 2021). European frog-bit rosettes can reproduce asexually through the formation of stolons and daughter rosettes, which can fragment and spread to new waterways and wetlands and form new colonies within a growing season (Catling et al. 2003). Asexual reproduction can also occur through the formation of turions, or vegetative buds. A single turion can form a rosette and produce ramets that cover a full square metre by the end of a single growing season (Catling et al. 2003). Established populations of mat-forming EFB in Saginaw Bay produced 1,537 (±780) turions per square metre(Cahill et al. 2021). European frog-bit rosettes, turions and seeds may spread throughout and between waterbodies by water flow and wave action, attached to boating and fishing equipment, through intentional planting and release from aquaria and water gardens and by waterfowl (Catling and Dore 1982, Catling et al. 2003, Ganie et al. 2016).

European frog-bit has become an issue of concern in the Great Lakes Region due to the perception it could impact recreational water use and wetland ecosystems. European frog-bit can be found in dense floating mats that have the potential to limit water flow and impede commercial and recreational use of waterways (Cook and Lüönd 1982, Catling et al. 2003). A high abundance of EFB can reduce light, nutrients and dissolved oxygen in the water column and, subsequently, negatively impact habitat quality and native species diversity (Catling et al. 1988, Catling et al. 2003, Johnson 2018, Monfils et al. 2021). The expanding distribution and potential negative effects of dense EFB populations have raised concern amongst researchers, managers and the general public in its invasive range.

The European Frog-bit Collaborative was established in autumn 2018 to improve coordination and collaboration amongst stakeholders and build consensus on the next steps for EFB management and research in Michigan. The EFB Collaborative identified historic and current EFB distribution as a priority information need for EFB management. Known distribution is critical for determining habitat suitability and the factors that drive EFB invasion, predicting future EFB spread and establishment and identifying high-priority areas for targeted EFB monitoring and management. The aggregated dataset has helped guide the development of objectives, work plans and priorities as part of the European Frog-bit Adaptive Management Framework (EFB AMF; Cahill and Monfils 2021). The EFB AMF is a comprehensive management plan that engages researchers, managers and community members aimed at controlling EFB and mitigating its effects in Michigan. The data-driven application of the EFB AMF is essential to the preservation of wetland ecosystems impacted by EFB and the values they provide. Additionally, the dataset has provided information for prioritisation of targeted surveying for EFB in Michigan.

To compile the aggregated EFB occurrence dataset, we accessed specimen-based and observation-based data across the invasive range of North America. We included occurrence records from herbaria, digital data aggregators and individual researchers. Our aggregation and curation efforts included duplicate identification and removal, georeferencing and data validation and standardisation. The final dataset contains 12,037 records, including 9,994 presence records and 2,043 absence records (Hansen et al. 2022). This dataset provides a baseline for the historic and current distribution of EFB in North America, which is the first step towards effective, data-driven management actions.

## General description

### Purpose

Coastal and inland aquatic ecosystems in the United States and Canada are threatened by the spread of invasive European frog-bit. We aggregated, cleaned and curated all available specimen- and observation-based occurrence records in the U.S. and Canada to create a comprehensive spatiotemporal occurrence dataset across the invasive range of EFB in North America. The dataset has been used to provide information for management planning for EFB in Michigan and will continue to be used for EFB distribution modelling and risk assessments throughout the Great Lakes Region. By making the dataset freely available for reuse, we provide a valuable data resource for researchers and managers to continue EFB management efforts in the Great Lakes and beyond.

## Project description

### Title

Aggregated occurrence records of invasive European frog-bit (*Hydrocharismorsus-ranae* L.) across North America.

### Study area description

We include all accessible North American occurrences (observation- and specimen-based) dating from 1932 to July 2021 in the dataset. The data represent occurrence records from two Canadian provinces (Ontario and Quebec) and thirteen U.S. states, ten of which have recorded EFB presence (Florida, Maine, Michigan, New Jersey, New York, Ohio, Pennsylvania, Vermont, Washington State and Wisconsin) and three of which have recorded absence only (Illinois, Indiana and Kentucky; Table 1).

## Sampling methods

### Sampling description

European frog-bit occurrence records were aggregated from digital data repositories, natural history collections and university researchers (Table 2). A total of 23,985 observation-based and specimen-based digital records were initially obtained; after data validation and duplicate removal, the total aggregated dataset contains 12,037 unique records. Specimen-based data in natural history collections were accessed through digital specimen data aggregators: Consortium of Midwest Herbaria (Consortium of Midwest Herbaria 2021), Global Biodiversity Information Facility (GBIF; GBIF.org 2021), Great Lakes Invasives Network (Great Lakes Invasives Network 2021) and Integrated Digitized Biocollections (iDigBio; iDigBio 2021). Absence records were available for some sets of data and make up 2,043 of 12,037 total records.

Absence records were contributed from two specific projects:

Central Hardwood Invasives Plant Network Joint Aquatic Invasive Species Survey (CHIP-N) - Regional effort launched in 2009 to survey invasive species in the central midwestern United States (https://www.rtrcwma.org/CHIP-N/). This project contributed 328 total absence records spanning Illinois, Indiana and Ohio.

Central Michigan University Herbarium (CMC) - Research funded in 2020 to assess EFB abundance and ecosystem impacts in Saginaw Bay, St. Clair Flats and Lake Erie in Michigan. Data were collected using standard methodologies (Monfils et al. 2021). This project contributed 2,827 total records, including 1,715 absence and 1,112 presence.

The total number of records by observation-based data provider or herbarium collection is given in Table 2. In cases where an observation-based record is housed by multiple digital repositories, the initial aggregator is cited. Observation-based records comprise 95% of total records (11,404 of 12,037 records). Specimen-based records comprise 83% of the data available up to 2000 (399 of 481 records) and 5% of records through July 2021 (633 of 12,037 records).

### Quality control

Field names and contents of raw data were standardised to ensure consistent capitalisation, spelling, grammar, taxon naming conventions and formatting to conform to Darwin Core standards, as described in the Darwin Core Quick Reference Guide (Darwin Core Maintenance Group 2020).

We identified and consolidated duplicate observation-based records held by multiple online repositories, retaining all record numbers to ensure each merged record would be traceable to every online source that houses it. We merged specimen-based records only when they were taken from the same specimen sheet (as verified by examining each original specimen image) or associated with an observation-based record. When specimens were known to be physical duplicates (separate individuals collected by the same collector at the same time and place) or when images were not available, specimen-based records were retained.

We standardised information in the eventDate and eventTime fields to conform to ISO 8601-1:2019 and recorded original dates in the verbatimEventDate field, as per Darwin Core standards (Wieczorek et al. 2012). Occurrences recorded with dates on 1 January were inconsistent with the known phenology of EFB in its invasive range. For these records, only the record year was retained.

One hundred and fifty digitised specimen images were provided by Agriculture and Agri-Food Canada's National Collection of Vascular Plants (DAO; Agriculture and Agri-Food Canada 2021). Image labels were transcribed to Darwin Core fields. They include data as early as 1936, in the first few years of EFB's invasive spread following its 1932 introduction.

Data were corrected and modified only to the extent necessary to improve clarity and usability; original content was maintained whenever possible. When locality strings were updated using associated data or linked records, original locality strings were retained in verbatimLocality, as per Darwin Core standards (Wieczorek et al. 2012). We corrected specimen sheet transcriptions and reference URLs where needed.

When provided, original geographic coordinates were retained. When no coordinates of any kind were available (290 records), we georeferenced records using the GEOLocate Web Application (Rios and Bart 2010) and following the guidance of Chapman and Wieczorek (2020). We noted decisions regarding placement of coordinate points and their associated uncertainty in locationRemarks and georeferenceRemarks. Seven records could not be georeferenced due to localities that were too broad or indeterminate, and we noted this in georeferenceRemarks.

## Geographic coverage

### Description

**Figure 1.**
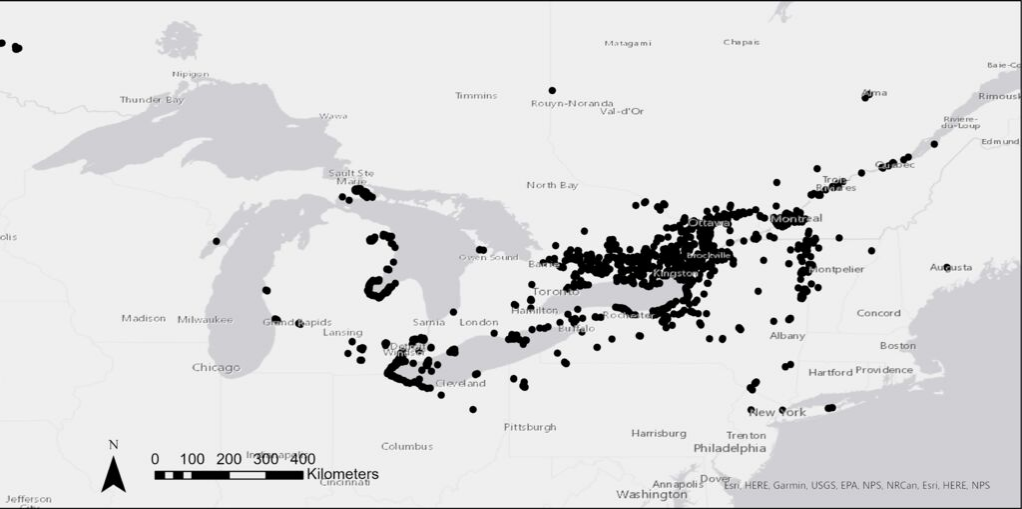
Presence records of European frog-bit in Canada and the United States. Occurrences in Washington State and Florida are not shown.

Records in the dataset span two Canadian provinces and thirteen U.S. states (Table 1, Fig. 1). Coordinates reflect the extent of presence records only.

### Coordinates

30.3207 and 49.7652 Latitude; -121.9681 and -69.8762 Longitude.

## Taxonomic coverage

### Description

This dataset contains records from a single species, European frog-bit (*Hydrocharismorsus-ranae* L.) Higher classifications were obtained from Integrated Taxonomic Information System (2021).

### Taxa included

**Table:** 

Rank	Scientific Name	
kingdom	Plantae	
class	Magnoliopsida	
order	Alismatales	
family	Hydrocharitaceae	
genus	*Hydrocharis*	
species	*Hydrocharismorsus-ranae* L.	

## Temporal coverage

### Notes

**Figure 2.**
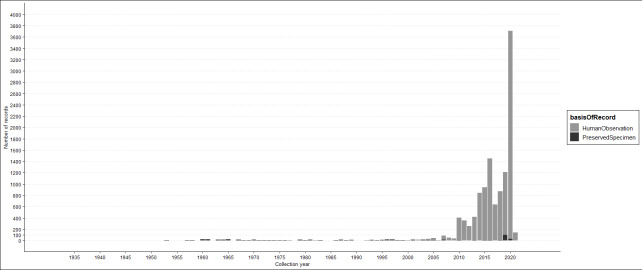
Temporal distribution of European frog-bit PreservedSpecimen and HumanObservation records.

The data range from 1932 to 29 July 2021 (Fig. 2). The increase in observation records after 2010 is consistent with the invasive spread of EFB and increased surveying effort across the Great Lakes Region.

## Usage licence

### Usage licence

Other

### IP rights notes

See individual records for usage rights.

## Data resources

### Data package title

**Aggregated occurrence records of invasive European frog-bit (*Hydrocharismorsus-ranae* L.) across North America.**

### Resource link

https://www.gbif.org/dataset/71454d8a-6e9c-49f5-bf37-353f9ad2e2b9

### Number of data sets

1

### Data set 1.

#### Data set name

Aggregated *Hydrocharismorsus-ranae* L. occurrence records.

#### Data format

Darwin Core Archive

#### Number of columns

89

#### Data format version

2021-07-15

#### Description

Data fields and contents have been standardised to 89 Darwin Core Archive columns. The following table lists column names and descriptions as they appear in the Darwin Core quick reference guide (Darwin Core Maintenance Group 2020). The dataset contains both presence and absence records of a single species, European frog-bit (*Hydrocharismorsus-ranae* L.) and is freely available to download (Hansen et al. 2022).

**Data set 1.** 

Column label	Column description
type	The nature or genre of the resource.
modified	The most recent date-time on which the resource was changed.
language	A language (or languages) of the resource.
license	A legal document giving official permission to do something with the resource.
rightsHolder	A person or organisation owning or managing rights over the resource.
accessRights	Information about who can access the resource or an indication of its security status.
bibliographicCitation	A bibliographic reference for the resource as a statement indicating how this record should be cited (attributed) when used.
references	A related resource that is referenced, cited or otherwise pointed to by the described resource.
institutionID	An identifier for the institution having custody of the object(s) or information referred to in the record.
collectionID	An identifier for the collection or dataset from which the record was derived.
datasetID	An identifier for the set of data. May be a global unique identifier or an identifier specific to a collection or institution.
institutionCode	The name (or acronym) in use by the institution having custody of the object(s) or information referred to in the record.
collectionCode	The name, acronym, coden or initialism identifying the collection or dataset from which the record was derived.
datasetName	The name identifying the dataset from which the record was derived.
ownerInstitutionCode	The name (or acronym) in use by the institution having ownership of the object(s) or information referred to in the record.
basisOfRecord	The specific nature of the data record.
informationWithheld	Additional information that exists, but that has not been shared in the given record.
dynamicProperties	A list of additional measurements, facts, characteristics, or assertions about the record. Meant to provide a mechanism for structured content.
occurrenceID	An identifier for the Occurrence (as opposed to a particular digital record of the occurrence). In the absence of a persistent global unique identifier, construct one from a combination of identifiers in the record that will most closely make the occurrenceID globally unique.
catalogNumber	An identifier (preferably unique) for the record within the dataset or collection. Note: If a catalogNumber did not exist, one was constructed from existing record identifiers.
recordNumber	An identifier given to the Occurrence at the time it was recorded. Often serves as a link between field notes and an Occurrence record, such as a specimen collector's number.
recordedBy	A list (concatenated and separated) of names of people, groups or organisations responsible for recording the original Occurrence. The primary collector or observer, especially one who applies a personal identifier (recordNumber), should be listed first.
recordedByID	A list (concatenated and separated) of the globally unique identifier for the person, people, groups or organisations responsible for recording the original Occurrence.
individualCount	The number of individuals represented present at the time of the Occurrence.
lifeStage	The age class or life stage of the biological individual(s) at the time the Occurrence was recorded.
reproductiveCondition	The reproductive condition of the biological individual(s) represented in the Occurrence.
establishmentMeans	Statement about whether an organism or organisms have been introduced to a given place and time through the direct or indirect activity of modern humans.
occurrenceStatus	A statement about the presence or absence of a Taxon at a Location.
preparations	A list (concatenated and separated) of preparations and preservation methods for a specimen.
disposition	The current state of a specimen with respect to the collection identified in collectionCode or collectionID.
associatedMedia	A list (concatenated and separated) of identifiers (publication, global unique identifier, URI) of media associated with the Occurrence.
associatedReferences	A list (concatenated and separated) of identifiers (publication, bibliographic reference, global unique identifier, URI) of literature associated with the Occurrence.
associatedSequences	A list (concatenated and separated) of identifiers (publication, global unique identifier, URI) of genetic sequence information associated with the Occurrence.
associatedTaxa	A list (concatenated and separated) of identifiers or names of taxa and their associations with the Occurrence.
otherCatalogNumbers	A list (concatenated and separated) of previous or alternate fully qualified catalogue numbers or other human-used identifiers for the same Occurrence, whether in the current or any other dataset or collection.
occurrenceRemarks	Comments or notes about the Occurrence.
fieldNumber	An identifier given to the event in the field. Often serves as a link between field notes and the Event.
eventDate	The date-time or interval during which an Event occurred. For occurrences, this is the date-time when the event was recorded.
eventTime	The time or interval during which an Event occurred.
startDayOfYear	The earliest integer day of the year on which the Event occurred (1 for 1 January, 365 for 31 December, except in a leap year, in which case it is 366).
endDayOfYear	The latest integer day of the year on which the Event occurred (1 for 1 January, 365 for 31 December, except in a leap year, in which case it is 366).
year	The four-digit year in which the Event occurred, according to the Common Era Calendar.
month	The integer month in which the Event occurred.
day	The integer day of the month on which the Event occurred.
verbatimEventDate	The verbatim original representation of the date and time information for an Event.
habitat	A category or description of the habitat in which the Event occurred
eventRemarks	Comments or notes about the Event.
continent	The name of the continent in which the Location occurs.
waterBody	The name of the water body in which the Location occurs.
island	The name of the island on or near which the Location occurs.
country	The name of the country or major administrative unit in which the Location occurs.
countryCode	The standard code for the country in which the Location occurs.
stateProvince	The name of the next smaller administrative region than country (state, province, canton, department, region, etc.) in which the Location occurs.
county	The full, unabbreviated name of the next smaller administrative region than stateProvince (county, shire, department, etc.) in which the Location occurs.
municipality	The full, unabbreviated name of the next smaller administrative region than county (city, municipality, etc.) in which the Location occurs. Do not use this term for a nearby named place that does not contain the actual location.
locality	The specific description of the place. Less specific geographic information can be provided in other geographic terms (higherGeography, continent, country, stateProvince, county, municipality, waterBody, island, islandGroup). This term may contain information modified from the original to correct perceived errors or standardise the description.
verbatimLocality	The original textual description of the place.
minimumElevationInMeters	The lower limit of the range of elevation (altitude, usually above sea level), in metres.
verbatimElevation	The original description of the elevation (altitude, usually above sea level) of the Location.
verbatimDepth	The original description of the depth below the local surface.
locationAccordingTo	Information about the source of this Location information. Could be a publication (gazetteer), institution or team of individuals.
locationRemarks	Comments or notes about the Location.
decimalLatitude	The geographic latitude (in decimal degrees, using the spatial reference system given in geodeticDatum) of the geographic centre of a Location. Positive values are north of the Equator, negative values are south of it. Legal values lie between -90 and 90, inclusive.
decimalLongitude	The geographic longitude (in decimal degrees, using the spatial reference system given in geodeticDatum) of the geographic centre of a Location. Positive values are east of the Greenwich Meridian, negative values are west of it. Legal values lie between -180 and 180, inclusive.
geodeticDatum	The ellipsoid, geodetic datum or spatial reference system (SRS) upon which the geographic coordinates given in decimalLatitude and decimalLongitude are based.
coordinateUncertaintyInMeters	The horizontal distance (in metres) from the given decimalLatitude and decimalLongitude describing the smallest circle containing the whole of the Location. Leave the value empty if the uncertainty is unknown, cannot be estimated or is not applicable (because there are no coordinates). Zero is not a valid value for this term.
verbatimCoordinates	The verbatim original spatial coordinates of the Location. The coordinate ellipsoid, geodeticDatum or full Spatial Reference System (SRS) for these coordinates should be stored in verbatimSRS and the coordinate system should be stored in verbatimCoordinateSystem.
verbatimLatitude	The verbatim original latitude of the Location. The coordinate ellipsoid, geodeticDatum or full Spatial Reference System (SRS) for these coordinates should be stored in verbatimSRS and the coordinate system should be stored in verbatimCoordinateSystem.
verbatimLongitude	The verbatim original longitude of the Location. The coordinate ellipsoid, geodeticDatum or full Spatial Reference System (SRS) for these coordinates should be stored in verbatimSRS and the coordinate system should be stored in verbatimCoordinateSystem.
verbatimCoordinateSystem	The coordinate format for the verbatimLatitude and verbatimLongitude or the verbatimCoordinates of the Location.
georeferencedBy	A list (concatenated and separated) of names of people, groups or organisations who determined the georeference (spatial representation) for the Location.
georeferencedDate	The date on which the Location was georeferenced.
georeferenceProtocol	A description or reference to the methods used to determine the spatial footprint, coordinates and uncertainties.
georeferenceSources	A list (concatenated and separated) of maps, gazetteers or other resources used to georeference the Location, described specifically enough to allow anyone in the future to use the same resources.
georeferenceVerificationStatus	A categorical description of the extent to which the georeference has been verified to represent the best possible spatial description.
georeferenceRemarks	Notes or comments about the spatial description determination, explaining assumptions made in addition or opposition to the those formalised in the method referred to in georeferenceProtocol.
identificationID	An identifier for the Identification (the body of information associated with the assignment of a scientific name). May be a global unique identifier or an identifier specific to the dataset.
identifiedBy	A list (concatenated and separated) of names of people, groups or organisations who assigned the Taxon to the subject.
identifiedByID	A list (concatenated and separated) of the globally unique identifier for the person, people, groups or organisations responsible for assigning the Taxon to the subject.
dateIdentified	The date on which the subject was determined as representing the Taxon.
identificationRemarks	Comments or notes about the Identification.
scientificName	The full scientific name, with authorship and date information if known.
kingdom	The full scientific name of the kingdom in which the taxon is classified.
class	The full scientific name of the class in which the taxon is classified.
order	The full scientific name of the order in which the taxon is classified.
family	The full scientific name of the family in which the taxon is classified.
genus	The full scientific name of the genus in which the taxon is classified.
taxonRank	The taxonomic rank of the most specific name in the scientificName.
vernacularName	A common or vernacular name.

## Figures and Tables

**Table 1.** Presence and absence records by state and province from the first record in North America in 1932 to 2021.

State/Province	Presence	Absence
Ontario, Canada	1596	0
Quebec, Canada	250	0
Florida, United States	1	0
Illinois, United States	0	75
Indiana, United States	0	92
Kentucky, United States	0	3
Maine, United States	2	0
Michigan, United States	6571	1715
New Jersey, United States	1	0
New York, United States	1394	0
Ohio, United States	74	158
Pennsylvania, United States	31	0
Vermont, United States	65	0
Washington, United States	8	0
Wisconsin, United States	1	0

**Table 2.** Number of observation-based and specimen-based records in the aggregated dataset by data provider.

	**Records**
**Observation-based data provider**	**11,404**
Central Michigan University Herbarium (CMC; Monfils 2021)	2,827
Early Detection and Distribution Mapping System (EDDMapS; EDDMapS 2021)	722
Global Biodiversity Information Facility (GBIF; GBIF.org 2021)	17
Literature-derived (Lamont et al. 2014)	1
iMapInvasives (iMapInvasives 2021)	835
iNaturalist (K 2021)	746
Midwest Invasive Species Information Network (MISIN; MISIN 2021)	6,131
United States Geological Survey Nonindigenous Aquatic Species Database (USGS NAS; U.S. Geological Survey 2021)	125
**Specimen-based data provider**	**633**
Agriculture and Agri-Food Canada National Collection of Vascular Plants (DAO)	150
Austin Peay State University Herbarium (APSC)	1
B.A. Bennett Herbarium (BABY)	1
Buffalo Museum of Science (BUF)	4
Canadian Museum of Nature (CMN)	15
Carnegie Museum of Natural History (CM)	3
Central Michigan University Herbarium (CMC)	130
Chico State Herbarium (CHSC)	1
Harvard University New England Botanical Club Herbarium (NEBC)	1
Herbier du Québec (QUE)	14
Indiana University Herbarium (IND)	2
Kent State University Herbarium (KE)	1
Marie-Victorin Herbarium (MT)	42
McGill University Herbarium (MTMG)	4
Miami University Willard Sherman Turrell Herbarium (MU)	7
Michigan State University Herbarium (MSC)	4
New York Botanical Garden Herbarium (NY)	5
New York State Museum (NYS)	11
Northern Kentucky University Herbarium (KNK)	1
Royal British Columbia Museum (RBCM)	3
Royal Ontario Museum Green Plant Herbarium (TRT)	17
Smithsonian National Museum of Natural History (US)	4
State University of New York, College at Oneonta Herbarium (SUCO)	1
Université Laval Herbier Louis-Marie (QFA)	80
University of Alberta Vascular Plant Herbarium (ALTA)	16
University of British Columbia Herbarium (UBC)	6
University of Calgary Herbarium (UAC)	2
University of California Riverside Plant Herbarium (UCR)	1
University of Connecticut George Safford Torrey Herbarium (CONN)	11
University of Georgia Herbarium (GA)	2
University of Idaho Herbarium (ID)	2
University of Illinois Herbarium (ILL)	1
University of Michigan Herbarium (MICH)	77
University of Notre Dame Greene-Nieuwland Herbarium (ND)	1
University of Texas Herbarium (TEX)	1
University of Vermont Pringle Herbarium (VT)	5
University of Washington Herbarium (WTU)	5
Wisconsin State Herbarium (WIS)	1
